# Genome-Wide Analysis of the Yeast Transcriptome Upon Heat and Cold Shock

**DOI:** 10.1002/cfg.301

**Published:** 2003-07

**Authors:** M. Becerra, L. J. Lombardía, M. I. González-Siso, E. Rodríguez-Belmonte, N. C. Hauser, M. E. Cerdán

**Affiliations:** 1 Dpto. Biología Celular y Molecular Universidad de La Coruña F. Ciencias Campus de La Zapateira s/n La Coruña 15075 Spain; 2 Molecular-Genetic Genome Analysis Group Deutsches Krebsforschungszentrum Heidelberg Germany; 3 Dpto. de Biología Celular y Molecular F. Ciencias.Universidad de A Coruña Campus de A Zapateira s/n A Coruña 15071 Spain

## Abstract

DNA arrays were used to measure changes in transcript levels as yeast cells responded to temperature shocks. The number of genes upregulated by temperature shifts from 30 ℃ to 37℃ or 45℃ was correlated with the severity of the stress. Pre-adaptation of cells, by growth at 37 ℃ previous to the 45℃ shift, caused a decrease in the number of genes related to this response. Heat shock also caused downregulation of a set of genes related to metabolism, cell growth and division, transcription, ribosomal proteins, protein synthesis and destination. Probably all of these responses combine to slow down cell growth and division during heat shock, thus saving energy for cell rescue. The presence of putative binding sites for Xbp1p in the promoters of these genes suggests a hypothetical role for this transcriptional repressor, although other mechanisms may be considered. The response to cold shock (4℃) affected a small number of genes, but the vast majority of those genes induced by exposure to 4 ℃ were also induced during heat shock; these genes share in their promoters *cis*-regulatory elements previously related to other stress responses.


**Comparative and Functional Genomics**
*Comp Funct Genom* 2003; **4**: 366–375.
Published online 18 July 2003 in Wiley InterScience (www.interscience.wiley.com). **DOI:** 10.1002/cfg.301

**Primary Research Paper**

### Genome-wide analysis of the yeast transcriptome upon heat and cold shock

M. Becerra[1], L. J. Lombardía[1], M. I. González-Siso[1], E. Rodríguez-Belmonte[1], N. C. Hauser[2] and M. E. Cerdán[1]*

[1] *Dpto. Biología Celular y Molecular, Universidad de La Coruña, F. Ciencias, Campus de La Zapateira s/n 15075, La Coruña, Spain*
[2] *Molecular-Genetic Genome Analysis Group, Deutsches Krebsforschungszentrum, Heidelberg, Germany*

**Correspondence to:*
*M. E. Cerdán, Dpto. de Biología Celular y Molecular, F. Ciencias. Universidad de A Coruña, Campus de A Zapateira s/n A Coruña 15071, Spain.*
*E-mail: bmanamrt@udc.es*

Received: 1 October 2002
Revised: 2 May 2003
Accepted: 22 May 2003

### Abstract

**DNA arrays were used to measure changes in transcript levels as yeast cells responded to temperature shocks. The number of genes upregulated by temperature shifts from 30 °C to 37 °C or 45 °C was correlated with the severity of the stress. Pre-adaptation of cells, by growth at 37 °C previous to the 45 °C shift, caused a decrease in the number of genes related to this response. Heat shock also caused downregulation of a set of genes related to metabolism, cell growth and division, transcription, ribosomal proteins, protein synthesis and destination. Probably all of these responses combine to slow down cell growth and division during heat shock, thus saving energy for cell rescue. The presence of putative binding sites for Xbp1p in the promoters of these genes suggests a hypothetical role for this transcriptional repressor, although other mechanisms may be considered. The response to cold shock (4 °C) affected a small number of genes, but the vast majority of those genes induced by exposure to 4 °C were also induced during heat shock; these genes share in their promoters *cis*-regulatory elements previously related to other stress responses. Copyright © 2003 John Wiley & Sons, Ltd.**

**Keywords:** **yeast; transcriptional profiling; heat shock; cold shock; pre-adaptation; stress response**

### Introduction

*Saccharomyces cerevisiae* is a good eukaryotic model for the study of gene expression on a global scale. The availability of the genome sequence of this microorganism has allowed the development of DNA arrays in which specific PCR products for each gene are used as targets for hybridization with cDNA obtained from mRNA isolated from cells exposed to different conditions. This technique is very useful for studying mechanisms that operate at the transcriptional level in order to adapt the cell to environmental changes. The effects caused by an increase in the growth temperature to 37 °C or 39 °C have been studied by several approaches, in relation to the cell wall integrity signalling pathway (Jung and Levin, 1999) and the stress response (Gasch *et al*., 2000; Causton *et al*., 2001). On the other hand, using cDNA subtraction screening, Zhang *et al*. (2001) identified five *Saccharomyces cerevisiae* genes whose expression was upregulated when the temperature was downshifted from 30 °C to 10 °C.

Here, using arrays on polypropylene membranes as previously described (Hauser *et al*., 1998), we report a genome-wide analysis of changes in yeast mRNA levels resulting from exposing the cells to heat shock (37 °C or 45 °C) or cold shock (4 °C). The effect of pre-adaptation to 37 °C, before the 45 °C shift, was also investigated. The selected conditions are more severe than those previously studied (Jung and Levin, 1999; Gasch *et al*., 2000; Causton *et al*., 2001; Zhang *et al*., 2001) and allow

Copyright © 2003 John Wiley & Sons, Ltd.

the identification of a larger set of regulated genes than has been previously reported.

### Materials and methods

### Strains and growth conditions

The haploid strain used in this study was FY73 (*Mat*$\alpha$*, ura3-52, his3*$\Delta$*200*), from which the diploid strain FY1679, used for the yeast sequencing project, is derived (Winston *et al.*, 1995).

The cells were grown at 30 °C in rich YPD media with 2% glucose until $OD_{600} = 0.8$. One fraction was shifted to 4 °C for 180 min (30 > 4 °C), another was shifted to 45 °C for 15 min (30 > 45 °C) and the third to 37 °C for 30 min. This last one was then divided into two; one half was left at 37 °C (30 > 37 °C) and the other was shifted to 45 °C for 15 min (30 > 37 > 45 °C). The times from the temperature shift until harvesting the cells were chosen according to previous data in the literature. Taking into account the transient nature of the transcriptional response, exposure to 37 °C was prolonged for only 30 or 45 min because the interval of maximal changes had been previously established as being 15–45 min (Gasch *et al.*, 2000; Causton *et al.*, 2001). The shift to 45 °C was shorter than the shift to 37 °C in order to obtain equivalent survival rates for cells in both conditions. The duration of the cold shift was established as 180 min because previous data on the induction of the LOT (low temperature responsive) genes were obtained at time intervals of 2–4 h (Zhang *et al.*, 2001).

### RNA isolations

Cells were harvested and immediately frozen in liquid nitrogen. They were then disrupted using a Micro-Dismembrator (B. Braun Biotech International). The resulting powder was mixed with TRIZOL Reagent (Life Technologies) and total RNA was extracted by the method of Chomczynski and Sacchi (1987).

### Probe generation

Probe generation was as described in Hauser *et al.* (1998). Briefly, 60 µg total RNA was annealed to oligonucleotide dT15 and used as a template to synthesize and radiolabel the corresponding first strand cDNA with 50 µCi of [$\alpha$-$^{33}$P]-dCTP (Amersham) using SuperScript II (Life Technologies). The reactions were carried out at 43 °C for 1 h, after which the RNA was hydrolysed with NaOH at 65 °C for 30 min. The probe was purified by isopropanol precipitation and the isotope incorporation was measured to check the efficiency of the reaction.

### Filters hybridizations, washings and stripping

Filters were prehybridized for 1 h at 65 °C in the hybridization mix: 5× SSC, 5× Denhardt's solution and 0.5% SDS. The probe was then denatured for 5 min at 100 °C, cooled quickly on ice and hybridized with the arrays overnight at 65 °C. On the following day two washes were carried out at hybridization temperature, for 5 and 20 min respectively, in 2× SSC 0.1% SDS. Filter regeneration was performed by pouring a boiling solution of 5 mM sodium phosphate (pH 7.5) and 0.1% SDS over the filters prior to their reuse.

### Signal quantification

The filters were exposed for 24 h to a storage phosphor screen and data collected using a PhosphorImager Scanning Instrument 425 (Molecular Dynamics). Signal quantification was performed with Array Vision software (Molecular Dynamics), which localizes over each array element a bounding circle fitted to the size of the DNA spot. Local area background was defined manually, placing 10 bounding circles throughout the filter. For each condition, the data from four independent hybridizations were analysed, using two different arrays and two different RNA samples. Therefore, a total of eight replica-spots per gene were analysed, since each array contained two replica-spots per gene.

### Statistical and computational analysis

Taking into account the numerous intrinsic variations of transcriptional profiling on arrays, an efficient statistical analysis (Beissbarth *et al.*, 2000) was performed to ensure the significance of the conclusions extracted from the data. The significance of the observed variations for each gene in two different conditions was assessed and classified by using two stringency criteria. The highly stringent 'min–max separation' is calculated by taking the minimum distance between

Copyright © 2003 John Wiley & Sons, Ltd.

all data points of the two conditions. The less stringent criteria, called 'standard deviation separation', is defined as the difference of the means of either data set diminished by one standard deviation. According to these stringency criteria, all data obtained were classified as being of high (min–max separation criteria), medium (standard deviation separation criteria) or low (the rest) statistical significance. Data quality assessment, normalization and statistical analysis were performed with the M-CHIPS software package (Fellenberg *et al.*, 2001, 2002) (**http://www.dkfz-heidelberg.de/tbi/services/mchips**) written in MATLAB. The complete transcript profiling data set is available as a table of the normalized results (**http://mips.gsf.de/proj/eurofan/eurofan_2/b2/dkfz/results_mce20.html**) with a colour code indicating the two significance criteria, and as raw data files (**http://mips.gsf.de/proj/eurofan/eurofan_2/b2/dkfz/results_mce50.txt**).

Correspondence analysis, an explorative computational method for the study of associations between variables, was carried out as previously described (Fellenberg *et al.*, 2001).

Additional cluster analysis to identify and represent groups of co-regulated genes was performed using the GeneCluster and TreeView software (Eisen *et al.*, 1998).

*In silico* analysis of promoters was carried out by the RSA tools facilities (**http://embnet.cifn.unam.mx/~jvanheld/rsa-tools/**) in a region extending up to −800 bp from the ATG of each gene considered.

### Results and discussion

The genome-wide analysis used to test the influence of high temperatures upon yeast transcription is a new approach compared with those previously reported in relation to environmental changes, including heat stress (Causton *et al.*, 2001; Gasch *et al.*, 2000; Jung and Levin, 1999). The analysis presented in our work addresses, however, two new perspectives, the problem of adaptation to heat shock by pre-exposure to less extreme conditions, and the inclusion and comparative analysis vs. cold shock.

### Effect of heat and cold shifts upon transcription

*Saccharomyces cerevisiae* cells in logarithmic phase growth were exposed to several environmental conditions caused by heat and cold shocks. These conditions involved shifts from 30 °C to 37 °C, 45 °C or 4 °C. In the case of heat-shock, the influence of pre-adaptation to 37 °C prior to the 45 °C shift was also studied. Total RNA was extracted and labelled cDNA derived from this was used in the hybridizations to polypropylene DNA arrays (Hauser *et al.*, 1998). Repeated hybridizations were performed, resulting in at least eight data sets each. Data were analysed using statistical procedures in order to select only the statistically significant changes, as described in Materials and methods.

The complete list for all genes included in the array as well as the complete transcript profiling data set is available in the form of lists of the normalized results, and raw data files, at the web addresses listed in Materials and methods.

For a global interpretation of transcriptional variation, the data were clustered using a bipole algorithm called correspondence analysis (CA), recently developed and applied to the analysis of microarray data (Fellenberg *et al.*, 2001). One big advantage of this procedure is the fact that both the experiments (hybridizations in each given condition) and the genes are clustered and presented in the same biplot. Points are depicted such that the sum of the distances of the points to their centroid is proportional to the value of the $X^2$ statistic of the data table. Distances among points (among genes or among hybridizations) are a measure of clustering. In contrast, the distance between a gene and a hybridization cannot be interpreted directly. The coloured lines in the biplot denote the direction of each condition and genes whose expression is correlated to a certain condition are located near to its direction line. The further away from the centroid (intersection of lines), the more pronounced is the association of the genes with this condition. Genes whose expression increases appear along the positive scale, while those decreasing appear on the opposite site of the centroid (negative scale).

Figure 1 shows the result of such a clustering applied to the data obtained in this study. Hybridizations related to the five conditions studied (four temperature shocks plus control) are represented by defined coloured squares (see the legend to Figure 1 for details), the medians of hybridization repeats by coloured circles, and expression levels of individual genes by black dots (the vast majority of genes without significant changes in expression are projected in the centroid and have

Copyright © 2003 John Wiley & Sons, Ltd. 

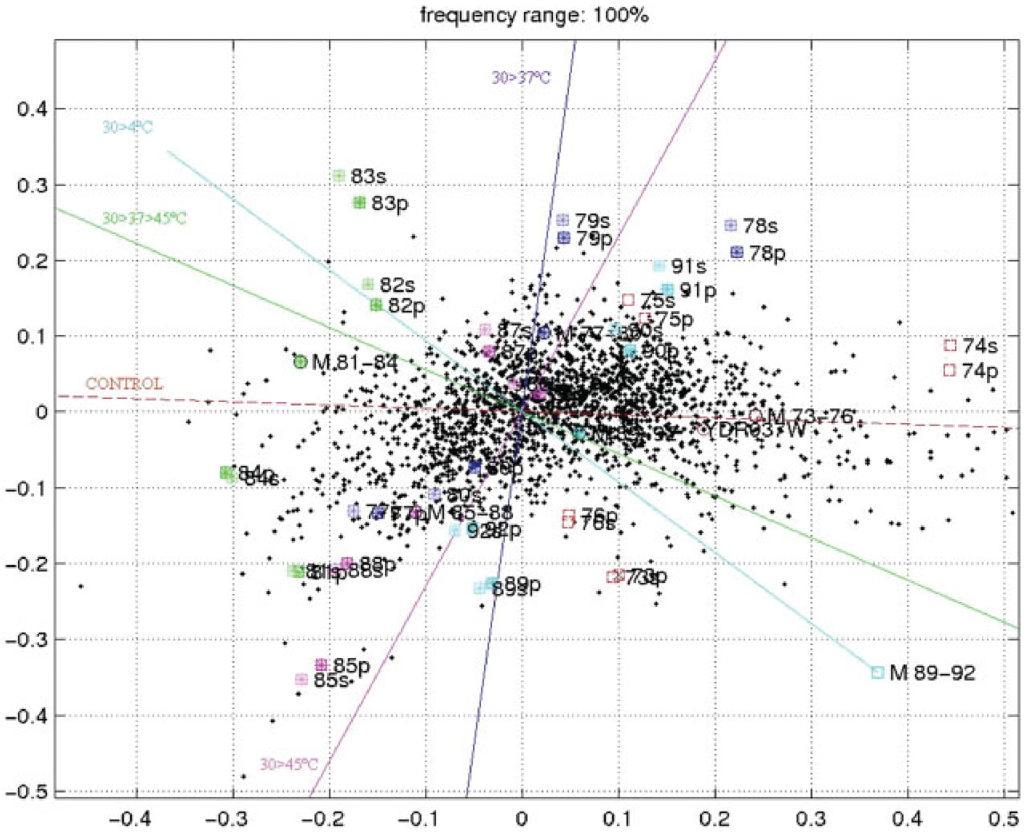

**Figure 1.** Biplot obtained by correspondence analysis of the normalized data from the *S. cerevisiae* transcriptome in different conditions characterized by heat and cold shocks. The coloured squares represent the respective experiments (hybridizations), each repeated four times and each showing the primary (p) and secondary (s) spots for the two repetitions of DNA probes present in these arrays. The coloured circles represent the hybridization medians. The black dots represent intensity signals for the individual genes. The majority of genes, equally transcribed in the five conditions and projecting over the centroid, have been removed to clear the figure. The biplot shows five different directions (coloured lines) corresponding to the four conditions analysed and the control (as indicated)

been removed for clarity). Each coloured line in the biplot defines the transcriptome under one specific condition. The biplot displayed in Figure 1 shows as many directions (coloured lines) as conditions analysed, thus revealing significant differences for the specific transcriptional response obtained in each condition. The conclusion is that the transcriptome profiles obtained in the four conditions analysed are different and specific to each shock.

Table 1 summarizes the number of genes whose expression changes in each of the conditions assayed. In constructing this table, only genes that change their expression according to the high and medium significance criteria established in the statistical analysis (Beissbarth *et al*., 2000) have been included. As explained in Materials and methods, highly significant variation between the specific condition and the control is attributed to those genes that fulfil the highly stringent 'min–max separation' criteria. Medium significance is attributed to those fulfilling the 'standard deviation separation' criteria.

As indicated in Table 1, the genes whose expression levels change (up or down) have low regulatory ratios between 1.5 and 3 and only a few show a more than three-fold change in relative expression. The following features are remarkable. First, the effect caused by temperature shock includes both activation and repression, and the number of downregulated genes is higher than the number of upregulated ones. This observation is in agreement with previous data from Gasch *et al*. (2000), who reported the same feature for genes regulated by the environmental stress response (ESR). Second,

Copyright © 2003 John Wiley & Sons, Ltd.

**Table 1.** The number of genes responding to each change

| | Nature of the temperature shock* | | | |
|---|---|---|---|---|
| | 30 > 37 °C | 30 > 45 °C | 30 > 37 > 45 °C | 30 > 4 °C |
| Downregulated genes $R > 1.5$ | 125 | 279 | 482 | 92 |
| $R > 2$ | 23 | 57 | 145 | 8 |
| $R > 3$ | 0 | 5 | 34 | 0 |
| Total | 148 | 341 | 661 | 100 |
| Upregulated genes $R > 1.5$ | 31 | 158 | 58 | 12 |
| $R > 2$ | 5 | 30 | 16 | 3 |
| $R > 3$ | 0 | 9 | 9 | 0 |
| Total | 36 | 197 | 83 | 15 |
| % of CER genes | 6 | 7.5 | 12.6 | |

* The nomenclature for each shock is the same as described in Materials and methods. *R*, regulatory ratio.

there are a larger number of genes related to the heat response than to the cold response. Third, in the heat response, the number of genes affected is correlated to the severity of the stress. Finally, pre-adaptation of cells by growth at 37 °C prior to the 45 °C shift causes a decrease in the number of upregulated genes. However, this effect is not observed for the downregulated genes. This difference may suggest that, after the heat shock, the genes whose transcription increases and those whose transcription decreases are controlled by separate regulatory circuits that respond differently to the pre-adaptation event.

### Transcription during heat shock

Figure 2 shows the expression profiles for cells exposed to the different temperature shifts. Single hierarchical clustering (Eisen *et al.*, 1998) was applied to the total set of genes whose transcript levels changed significantly in at least one of the heat shock conditions. The fold change is represented by the intensity of the colour (red for activation, green for repression). Most of the genes whose expression is up/downregulated in one condition also have the same response, although to a lesser extent, in the other conditions (clusters C and F). There are however, several clusters consisting of small numbers of genes that show an opposite response in one of the assayed conditions (A, B, D, E, G). The distribution of the functional categories of the genes in clusters C and F is also shown in Figure 2. The names of the genes in each cluster are given in the supplementary data **http://www.interscience.wiley.com/jpages/1531–6912/sites.html**.

Cluster C is composed of 567 genes whose expression diminishes in at least one of the heat conditions tested. A total of 382 of these genes (67%) are of unknown function. In the group of genes of known function, some functional categories are more strongly represented; these include genes related to metabolism, cell growth and division, transcription, ribosomal proteins and other genes related to protein synthesis and destination. These data are in agreement with previous results (Gasch *et al.*, 2000; Causton *et al.*, 2001). These results indicate that this downregulation is a very general response to heat which has not yet been considered in detail, but which probably serves to slow cell growth and division and to redirect energy resources to overcome the stress created by exposure to high temperature.

In a search for regulatory sequences that could be implicated in the general downregulation described above, the promoters of the genes included in cluster C (567 genes) were analysed using the RSA tools facilities to look for common conserved sequences. We also analysed the subset of genes that are downregulated, with $R > 2$, after the shift to 45 °C, with or without pre-adaptation to 37 °C. No statistically significant consensus was found in either of these analyses.

The existence of a general downregulation of transcription after heat shock is clearly depicted in the array data (Table 1, Figure 2). This downregulation principally affects functional categories related to protein synthesis and destination, as well as cell growth and division; in particular, ribosomal protein (RP) genes are highly represented. It has been reported that transcription of RP genes

Copyright © 2003 John Wiley & Sons, Ltd. 

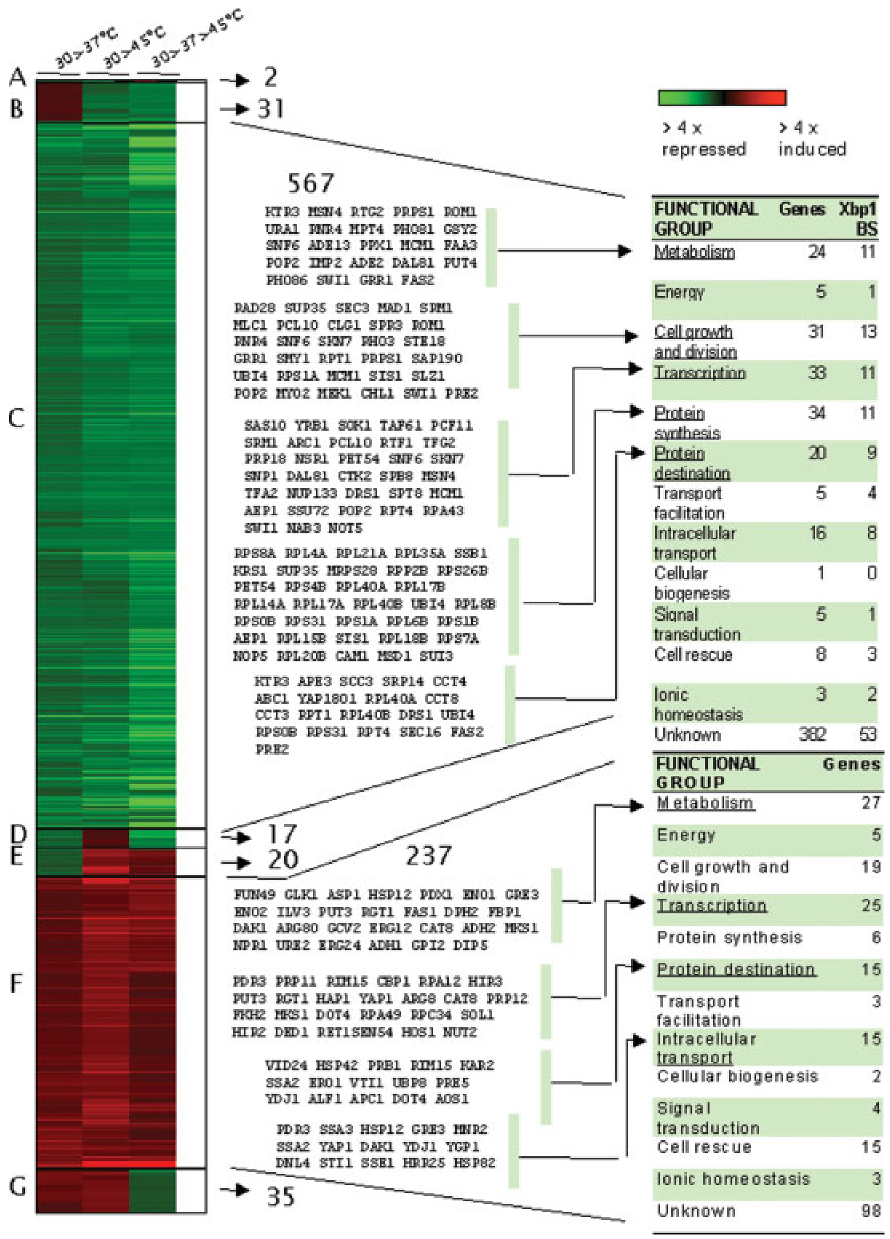

**Figure 2.** Clustering of the genes regulated by heat shock, according to Eisen *et al.* (1998). Green, downregulation; red, upregulation. The intensity of the colour is proportional to the regulation ratio. The distribution of functional categories (according to MIPS) of the genes, including the major clusters C and F, is also shown (right panel). The column headed Xbp1BS shows the number of promoters with putative binding sites for Xbp1p in each category can account for up to one-half of the RNA polymerase II-mediated transcription initiation events in the cell (Warner, 1999) and, therefore, this transcriptional decay could be a strategy to save energy, in order to develop molecular mechanisms for survival. We were unable to find a regulatory signal common to the promoters of those genes whose transcription diminishes after heat shock. This suggests a complex response that is probably the result of several mechanisms. This general downregulation could be an indirect effect of degradation, during heat shock, of mRNA or proteins

Copyright © 2003 John Wiley & Sons, Ltd. 

implicated in the transcription of these genes; in this sense, the mRNA levels of several general transcription factors, i.e. TAF61, TFG2, TFA2 or RNA polymerase II regulators, such as Ctk2, are downregulated after heat shock and their deficit could cause a general effect upon the transcription of many genes.

On the other hand, we also looked for putative regulatory sites binding the transcriptional repressor Xbp1p. The expression of *XBP1* is nearly undetectable under normal growth conditions but is strongly upregulated when cells are heat shocked, starved of glucose or treated with reagents causing osmotic shock, oxidative stress or DNA damage (Mai and Breeden, 1997). The number of promoters (defined as from −800 to +1 upstream sequence) having putative Xbp1p binding sites (with two substitutions allowed) in each of the functional groups of cluster C is shown in Figure 2.

The connection between downregulation and the control of cell growth and division is of particular interest. It has been reported that the $G_1$ cyclin genes *CLN1* and *CLN2* are transiently downregulated in heat-shocked cells (Rowley *et al.*, 1993) and that the *CLN1* promoter contains binding sites for Xbp1p, a stress-upregulated transcriptional repressor of the Swi4/Mbp1 family (Mai and Breeden, 1997). Analysis of the promoters of the 31 genes of cluster C that are functionally related to cell growth and division (Figure 2) showed that 13 contain the consensus sequence for Xbp1p binding (GNCTCGARGM). Besides this, the presence of *cis* sites for Xbp1p binding was also observed in the promoters of other genes that are co-regulated, like those related to protein synthesis and destination, transcription and metabolism.

Cluster F includes 237 genes whose expression is upregulated during the heat shifts and the more highly represented functional categories include, as expected, several chaperones and heat-shock proteins (i.e. Hsp12, Hsp82, Ssa3, Ydj1) but also a number of genes related to lipid metabolism (i.e. *ERG12, ERG24, FAS1, GPI2*). This result is in agreement with the recent hypothesis that lipid modifications are a major contributor to the induced heat and salt tolerance of yeast cells (Mahua *et al.*, 2000).

Among the genes related to specific transcriptional regulation, *YAP1* is also upregulated. The upregulation *of YAP1* by heat shock suggests a connection with the regulatory circuits of oxidative stress. Other supports for this connection come from the finding that expression of *GSH1* and *GSH2,* which code for enzymes of the glutathione biosynthetic pathway, are induced by oxidative stress and heat-shock in a Yap1p-dependent manner (Sugiyama *et al.*, 2000).

### The effect of pre-adaptation to 37 °C

We have previously shown (Table 1) that the intensity of the response is correlated to the intensity of the heat shock. Only 31 genes are upregulated by a shift to 37 °C for 30 min, while 158 are upregulated by a shift to 45 °C for 15 min. Moreover, not all of the genes that are upregulated under the first condition are also upregulated under the second. Therefore, there is a selective dependence between the upregulated genes and the intensity/duration of the heat shock.

In order to analyse the effect of pre-adaptation by growth at 37 °C, we have considered the interrelationship among genes that are upregulated by heat shock with high and medium statistical significance and ratios higher than 1.5 (Figure 3). The vast majority of genes that are upregulated at 45 °C (108 of 158) show no change if there is a pre-adaptation to 37 °C. *In silico* analysis of the promoters of these 108 genes responding to the pre-adaptation does not reveal any apparent common *cis*-regulatory signal. However, in the pool of genes that are upregulated independently of the existence of pre-adaptation, 31 of 34 have between 1 and

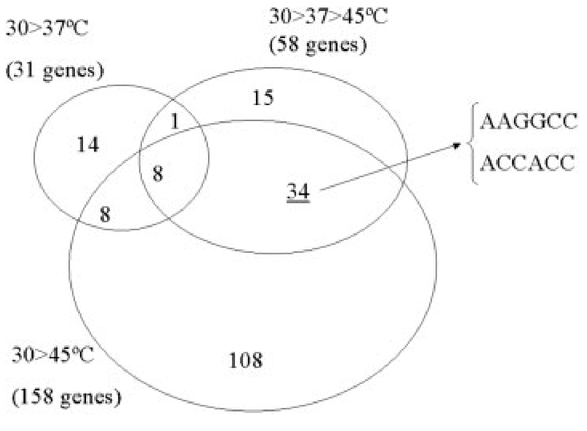

**Figure 3.** Venn diagram showing the summary of ORFs upregulated by heat shock and the effects of pre-adaptation to 37 °C

Copyright © 2003 John Wiley & Sons, Ltd. 

12 copies of the two consensus sequences AAG-GCC and ACCACC (with a significance of 0.8 and allowing only one substitution). These consensus sequences have not been previously identified as binding sites for known transcriptional factors.

### Correlation between heat and other stress responses

Among the genes downregulated or upregulated by the assayed heat shocks it is possible to find genes which have been classified as common environmental response (CER) genes, which are not specific for the response to temperature, but rather they take part in a general response to very diverse stimuli such as temperature, oxidation, nutrient availability, pH and osmolarity (Causton *et al*., 2001). In our analysis (Table 1), CER genes represent 6% of the genes upregulated by the shift to 37 °C, and 7.5% of the genes upregulated by the shift to 45 °C, but this last value increases to 12.6% if the shift to 45 °C is produced after pre-treatment at 37 °C. It has been hypothesized (Causton *et al*., 2001) that CER genes could explain the phenomena of tolerance and cross-protection, in which pre-treatment of cells with a mild environmental change provides protection against a more severe one. In the analysis of upregulated genes, the CER genes are more highly represented in the shift 30 > 37 > 45, which is a sort of progressive adaptation to higher temperature, than in the other shifts, and therefore our results give experimental support to this hypothesis.

### Correlation between heat and cold shocks

As shown in Table 1, the response to cold shock affects fewer genes than the response to heat shock. If the regulation ratio is set at a numerical value of

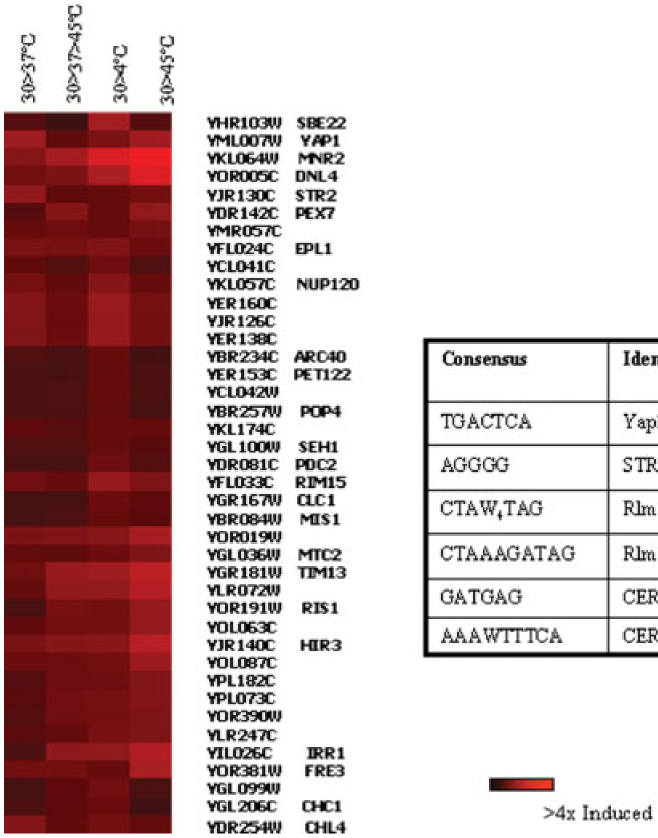

| Consensus | Identification | Number of genes |
|---|---|---|
| TGACTCA | Yap1p binding | 1 |
| AGGGG | STRE element | 38 |
| CTAW$_4$TAG | Rlm1p binding | 17 |
| CTAAAGATAG | Rlm1p binding | 3 |
| GATGAG | CER signal | 38 |
| AAAWTTTCA | CER signal | 30 |

**Figure 4.** Clustering of the genes upregulated by heat and cold shocks, according to Eisen *et al.* (1998). The intensity of the colour is proportional to the regulatory ratio. The panel on the right summarizes the number of genes in this cluster, which contain in their promoters known regulatory signals previously related to other stress responses

Copyright © 2003 John Wiley & Sons, Ltd. 

2 and only changes with high or medium statistical significance are considered, only eight genes (*YMR100W, SMY1, PMT3, APE3, URA2, SEC7, RPS4B, ANP1*) are expressed at lower levels after the cold shift. In the same conditions, only three genes are expressed at higher levels (*YJR126C, YER138C, IRR1*) and their function is unknown. The five low temperature-responsive (*LOT*) genes identified by cDNA subtraction screening (Zhang *et al.*, 2001) and the *TIP* and *TIR* genes, expressed during hypoxia and cold shock (Kondo and Inouye 1991; Kowalski *et al.*, 1995; Donzeau *et al.*, 1996), do not respond to the cold shift in our experimental conditions. Basically, the conditions previously reported (10 °C) and ours (4 °C) differ in the intensity of the stress. Although all of these genes are induced at 10 °C, it is likely that the exposure to 4 °C slows down the cellular machinery even beyond the allowed limit for induction of the cold tolerance response. Other factors that could explain the observed discrepancies may be the transient nature of the cold response and the stringency selected for the statistical analysis.

Many of the genes that respond to cold shift at 4 °C in our screening also respond to heat. Figure 4 shows the results of clustering cold- and heat-responsive genes altogether. In this case 47 genes upregulated by cold shock with regulatory ratios >1.5 and with high, medium or low statistical significance have been considered for the cluster. The results obtained show that 40 of these 47 genes are also upregulated by heat shock.

We performed an *in silico* analysis of the promoters of these 40 genes that are co-induced by cold and heat shocks, looking for *cis* signals for binding of Yap1p (TGACTCA), Rlm1p (CTA[T/A]$_4$TAG or CTAAAGATAG), or the STRE (AGGGG) or CER consensus (GATGAG or AAA (A/T)TTTTCA), and the results are shown in Figure 4. STRE elements and CER signals, recently defined by Causton *et al.* (2001), are a common feature in these promoters. The principal conclusion is that genes in this cluster share regulatory signals that are specific, not for the cold-response, but for general stress responses.

### Acknowledgements

Research in our laboratory was supported by the EUROFAN II project and Grants BMC2000-0117 (MCT, Spain) and PGIDT02TALI10301PR (XUGA, Spain). During 1999, L.-J.L.F. was the recipient of a post-doctoral fellowship from Universidad de La Coruña, XUGA (Spain). We are grateful to M. Vingron and K. Fellenberg (Heidelberg) for close collaboration and provision of software for data analyses. We are also grateful to the other members of the B2 consortium from the EUROFAN II project for cooperative and interactive support, especially to J. D. Hoheisel.

### References

Beissbarth T, Fellenberg K, Brors B, *et al.* 2000. Processing and quality control of DNA array hybridization data. *Bioinformatics* **16**: 1014–1022.

Causton HC, Ren B, Koh SS, *et al.* 2001. Remodelling of yeast genome expression in response to environmental changes. *Mol Biol Cell* **12**: 323–337.

Chatterjee MT, Khalawan SA, Curran BPG. 2000. Cellular lipid composition influences stress activation of the general stress response element (STRE). *Microbiology* **146**: 877–884.

Chomczynski P, Sacchi N. 1987. Single-step method of RNA isolation by acid guanidinium thiocyanate phenol chloroform extraction. *Anal Biochem* **162**: 156–159.

Donzeau M, Bourdineaud JP, Lauquin GJ. 1996. Regulation by low temperatures and anaerobiosis of a yeast gene specifying a putative GPI-anchored plasma membrane protein. *Mol Microbiol* **20**: 449–459.

Eisen MB, Spellman PT, Brown PO, Botstein D. 1998. Cluster analysis and display of genome-wide expression patterns. *Proc Natl Acad Sci USA* **95**: 14 863–14 868.

Fellenberg K, Hauser NC, Brors B, *et al.* 2001. Correspondence analysis applied to microarray data. *Proc Natl Acad Sci USA* **98**: 10 781–10 786.

Fellenberg K, Hauser NC, Brors B, *et al.* 2002. Microarray data warehouse allowing for the statistical analysis of experiment annotations. *Bioinformatics* **18**: 423–433.

Gasch AP, Spellman PT, Kao CM, *et al.* 2000. Genomic expression programs in the response of yeast cells to environmental changes. *Mol Biol Cell* **11**: 4241–4257.

Hauser NC, Fellenberg K, Gil R, *et al.* 2001. Whole genome analysis of a wine yeast strain. *Comp Funct Genom* **2**: 69–79.

Hauser NC, Vingron M, Scheideler M. 1998. Transcriptional profiling on all open reading frames of *Saccharomyces cerevisiae*. *Yeast* **14**: 1209–1221.

Jung US, Levin DE. 1999. Genome-wide analysis of gene expression regulated by the yeast cell wall integrity-signalling pathway. *Mol Microbiol* **34**: 1049–1057.

Kondo K, Inouye M. 1991. Cold-induction of yeast NSR1 protein and its role in pre-rRNA processing. *J Biol Chem* **267**: 16 259–16 265.

Kowalski LR, Kondo K, Inouye M. 1995. Cold-shock induction of a family of TIP1-related proteins associated with the membrane in *Saccharomyces cerevisiae*. *Mol Microbiol* **15**: 341–353.

Mai B, Breeden L. 1997. Xbp1, a stress-upregulated transcriptional repressor of the *Saccharomyces cerevisae* Swi4/Mbp1 family. *Mol Cell Biol* **17**: 6491–6501.

Copyright © 2003 John Wiley & Sons, Ltd. 

Rowley A, Johnston GC, Butler B, *et al*. 1993. Heat shock-mediated cell cycle blockage and $G_1$ cyclin expression in the yeast *Saccharomyces cerevisiae*. *Mol Cel Biol* **13**: 1034–1041.

Sugiyama K, Izawa S, Inoue Y. 2000. The Yap1-dependent induction of glutathione synthesis in heat shock response of *Saccharomyces cerevisiae*. *J Biol Chem* **19**: 15 535–15 540.

Warner J. 1999. The economics of ribosome biosynthesis in yeast. *Trends Biochem Sci* **24**: 437–440.

Winston F, Dollard C, Ricupero-Hovasse SL. 1995. Construction of a set of convenient *Saccharomyces cerevisiae* strains that are isogenic to S288C. *Yeast* **11**: 53–55.

Zhang L, Ohta A, Horiuchi H, Takagi M, Imai R. 2001. Multiple mechanisms regulate expression of low temperature responsive (LOT) genes in *Saccharomyces cerevisiae*. *Biochem Biophys Res Commun* **283**: 531–535.

Copyright © 2003 John Wiley & Sons, Ltd. 
